# Atmospheric muography for imaging and monitoring tropic cyclones

**DOI:** 10.1038/s41598-022-20039-4

**Published:** 2022-10-06

**Authors:** Hiroyuki K. M. Tanaka, Jon Gluyas, Marko Holma, Jari Joutsenvaara, Pasi Kuusiniemi, Giovanni Leone, Domenico Lo Presti, Jun Matsushima, László Oláh, Sara Steigerwald, Lee F. Thompson, Ilya Usoskin, Stepan Poluianov, Dezső Varga, Yusuke Yokota

**Affiliations:** 1 University of Tokyo, Tokyo, Japan; 2 International Virtual Muography Institute (VMI), Global, Tokyo, Japan; 3 Durham University, Durham, UK; 4 Kerttu Saalasti Institute, University of Oulu, Oulu, Finland; 5 Muon Solutions Oy Ltd, Pyhäsalmi, Finland; 6 Arctic Planetary Science Institute, Rovaniemi, Finland; 7 University of Catania, Catania, Italy; 8 Istituto Nazionale di Fisica Nucleare, Catania, Italy; 9 University of Sheffield, Sheffield, UK; 10 Geoptic Ltd, Warnborough, UK; 11 Sodankylä Geophysical Observatory and Space Physics and Astronomy Research Unit, University of Oulu, Oulu, Finland; 12 Space Physics and Astronomy Research Unit, University of Oulu, Oulu, Finland; 13 Wigner Research Centre for Physics, Budapest, Hungary; 14 Universidad de Atacama, Copiapò, Chile

**Keywords:** Atmospheric science, Particle physics, Experimental particle physics, Climate sciences, Physics

## Abstract

Large-scale solid bodies on Earth such as volcanoes and man-made pyramids have been visualized with solid earth muography, and the recently invented technique, acqueous muography, has already demonstrated its capability to visualize ocean tides and tsunami. In this work, atmospheric muography, a technique to visualize and monitor the vertical profile of tropic cyclones (TCs) is presented for the first time. The density distribution and time-dependent behavior of several TCs which had approached Kagoshima, Japan, has been investigated with muography. The resultant time-sequential images captured their warm cores, and their movements were consistent with the TC trails and barometric pressure variations observed at meteorological stations. By combining multidirectional muographic images with barometric data, we anticipate that muography will become a useful tool to monitor the three-dimensional density distribution of a targeted mesoscale convective system.

## Introduction

Mesoscale convective systems (MCSs) are organized groupings of meteorological systems that involve the most massive convective storm types^1^. These are storms defined as thunderstorms in the tropics^1–3^ and mid-latitudes^4^. They can span thousands of square kilometers. Storms qualifying as MCSs are major contributors to extreme precipitation^1^ and meteotsunamis^4^. There have been many previous papers that have discussed the relationship between MCS and precipitation, including those in the tropics^5–7^. However, even though researchers have identified the organized deep convection patterns in tropical MCS, characterization of each event is complex, being linked to the surrounding environment and/or to the MCS’s internal dynamics and microphysics^8^. These factors influence the development of these deep convective systems^9^ in somewhat mysterious ways.

Both tropical and warmer midlatitude MCSs are generated in response to atmospheric convective instability coupled with widespread and heavy rainfall. Stable and slow-moving MCSs frequently cause flooding, hail, strong winds, and tornadoes. Although state-of-the-art models are not yet able to explicitly represent convective precipitation due to their coarse grid spacings (i.e. 12/100 km at regional scale, a new model based on a 4 km grid spacing cannot effectively represent MCSs^1^, there are two hypotheses for the creation of MCSs. The first hypothesis is that the MCS’s convective cloud is generated by the condensation that occurs in nonhydrostatic buoyant upward air currents, where parcels of warm and moist air are accelerated upward^2^. These air currents are thought to subsequently generate in-cloud turbulence as they rise, and after being decelerated, this motion will eventually stop and spread out laterally at a level of neutral buoyancy^10^. Another traditional hypothesis of MCS generation surmises that an upward air motion drives the layers of air to adapt a deep slanting, ascending motion^11^^.^ According to this theory, the storm is characterized by a pressure reduction in the middle height of the MCS. Wind shear^3–5^ and density profile^6^ are the primary and essential parameters for buoyancy-driven convection of the atmosphere, and thus, understanding the pressure/density anomalies associated with MCSs can also be helpful in order to predict its mechanism. Regardless of which hypothesis about the formation of MCSs is ultimately found to be correct, it is evident that monitoring MCS pressure/density anomalies would bring researchers closer to creating accurate early warnings of MCS-associated hazards such as meteotsunamis^4^. Muography has been successfully tested in monitoring rapid sea level changes produced by meteostunamis within Tokyo Bay^12^, which could be an early warning in case of tsunamis or meteotsunamis.

Quantifying the mesoscale, and in particular, meso-beta (2–200 km) and meso-gamma (< 2 km) scale time-dependent pressure variations of the terrestrial atmosphere, would enable us to recognize the mechanisms of MCS, and muography has the capacity to be one of the solutions which could provide real-time mapping of the MCS associated pressure variations. Muography is similar to x-ray imagery, but instead of x-rays, it utilizes the strong penetration capability of high-energy muons (> a few tens of GeV) and their relativistic time-dilation effect. By detecting the number of muons that pass through gigantic bodies, like the mass of an entire or portion of an MCS, the internal spatial distribution of its density can be determined; this distribution can be mapped with muography by identifying where these muons passed through the object and subsequently creating a plot of the number of penetrating muons on a 2-dimensional plane. The origin of these high-energy muons is galactic cosmic rays (GCR) which are accelerated by supernovas and their remnants in our galaxy^13^. The GCRs mainly consist of nuclei of hydrogen and helium, though other are present in the composition. These charged particles are generally accelerated to almost the speed of light (10^–1^–10^11^ GeV), and spend millions of years in average in diffusive propagation in the interstellar magnetic field. This leads to the isotropic angular distribution of their momentum. In application to muography, it is reasonably safe to assume GCRs as isotropic at the entrance to the Earth’s atmosphere. Energetic GCRs are able to induce cascades of nuclear reactions in air, where multiple secondary particles are produced, generally classified as the hadronic, electromagnetic, muonic components and so-called atmospheric neutrinos. This work is focused at the muonic component (muons and antimuons) of cosmic-ray cascades in the atmosphere.

Muography takes advantage of the characteristics of the muon, particularly its penetrative nature and universality, for a wide variety of applications on Earth, including visualizing the internal structure of targeted gigantic solid earth and aqueous bodies^14–43^; its characteristics are also ideal for the application of visualizing the atmosphere. Solid muography (visualizing interiors of large-scale solid bodies on Earth) was invented by Luis Walter Alvarez and his group in 1968^14^, aqueous muography (visualizing and monitoring large-scale liquid bodies on Earth such as seas and lakes) was invented by Hiroyuki K.M. Tanaka and his group in 2021^43^. In this paper, the development of atmospheric muography (visualizing and monitoring large-scale gas bodies on Earth such as tropic cyclones) and its performance are presented. Ideas of this kind were proposed recently^44^ but not developed further. In their work, vertical muons were measured to trace temporal variations in the atomospheric pressure. As a result, vertically integrated profiles were generated as a function of latitude and altitude. In the current work, on the other hand, by utilyzing near-horizontal muons, imaging the vertical profile of the distant tropic cyclones was attempted. We report that a new muography imaging method could be used to two-dimensionally project density structures to render an image of TC (tropical cyclone) associated pressure anomaly variations.

## Results

### Vertical muon flux versus atmospheric pressure

**Figure 1:**
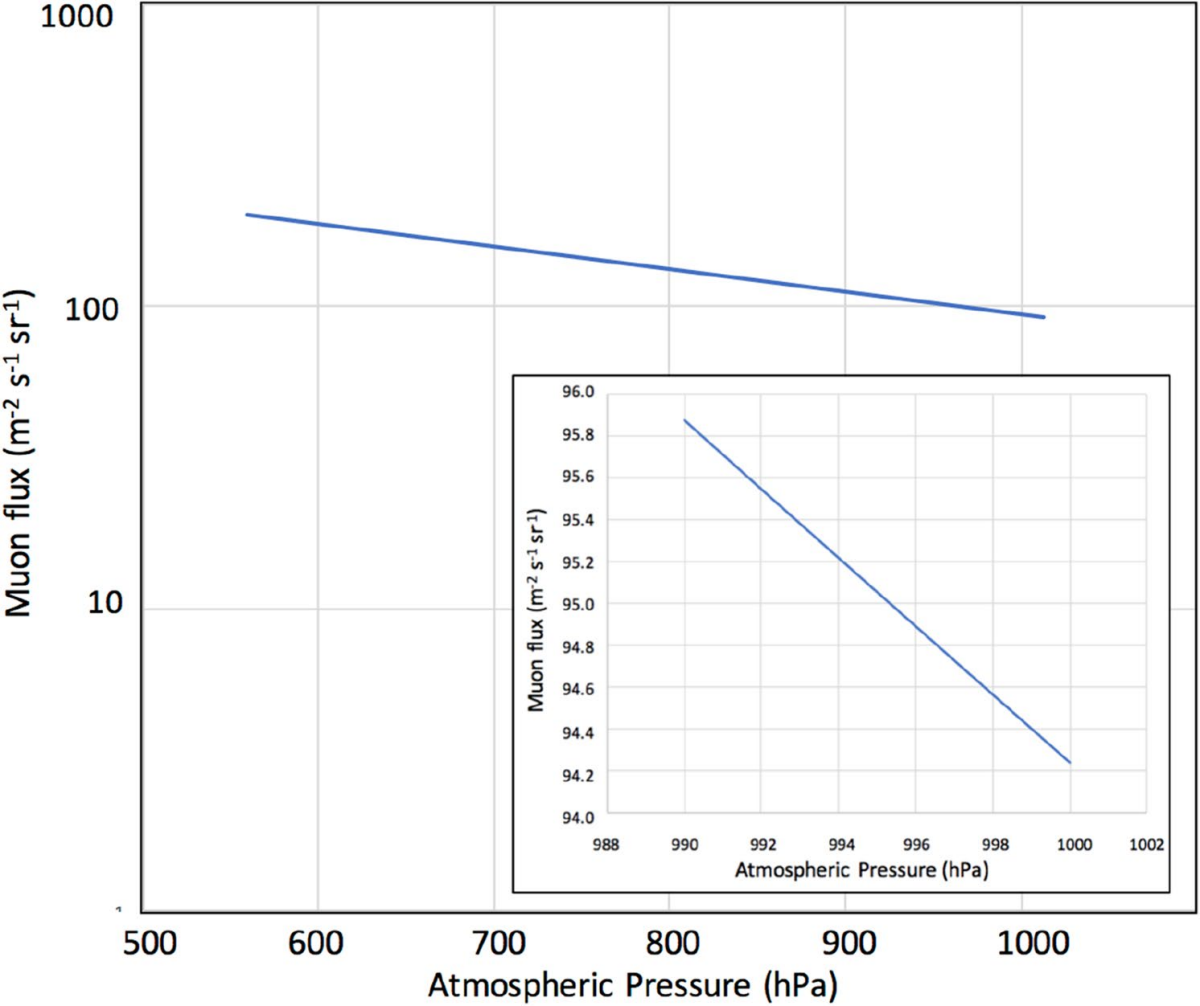
Vertical muon flux (N) versus atmospheric pressure (*ρ*). Inset illustrates the atmospheric pressure within the range between 990 and 1000 hPa.

Cosmic-ray muons are produced in interactions of primary cosmic rays and ambient atoms in the Earth’s atmosphere, and most muons (both µ^+^ and µ^−^) are generated around the tropopause (an atmospheric depth of 100 g cm^−2^). Figure 1 shows the relationship between the vertical muon flux (*N*) at sea level and the sea-level atmospheric pressure (*ρ*) on Earth^45^. If the integral mass of matter (e.g., in g cm^−2^ or hPa) along the muon path increases, the muons lose more energy before reaching sea level and thus, the probability that either the muon stops or decays increases. This relationship can be approximated to be a linear relationship within the atmospheric pressure range between 990 and 1000 hPa. Generally, if the pressure drops by 1%, the muon flux increases by ~ 2%. For horizontally arriving muons, since they traversed the atmosphere with a thickness of 36,000 g cm^−2^, an increase in the average mass along the muon path by 1% increases the minimum muon energy required to arrive at the ground surface by 1 GeV (an increase from 106 to 107 GeV)^46^. This reduces the horizontal muon flux from 4.5 to 4.3 m^−2^ s^−1^ sr^−1^ (~ 5%). Consequently, if the barometric pressure drops uniformly by 10 hPa throughout the upper hemisphere, the muon flux increases 2–5% depending on the muon's arrival angles.

### Observations

**Figure 2:**
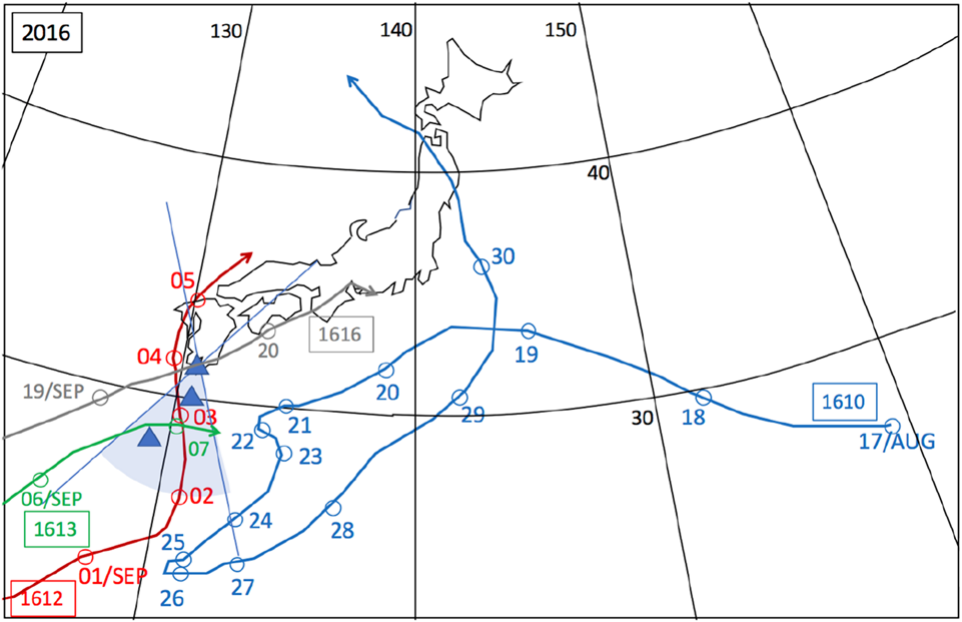
Meteorological history of tropical cyclones T-1610, T-1612, T-1613, and T-1616^48^. Black numbers next to the coordinate grid indicate the latitude and longitude. Blue, red, green, and grey numbers indicate the date of the positions of T-1610, T-1612, T-1613, and T-1616, respectively. Triangular symbols indicate the atmospheric pressure gauge stations (from north to south, Kagoshima, Yakushima and Naze stations). The azimuthal viewing angle of the Kagoshima Muograph (KM) is also indicated (shaded area). HKMT drew the map with Microsoft PowerPoint software and HKMT drew the image with Microsoft PowerPoint software and holds the copyright.

**Figure 3:**
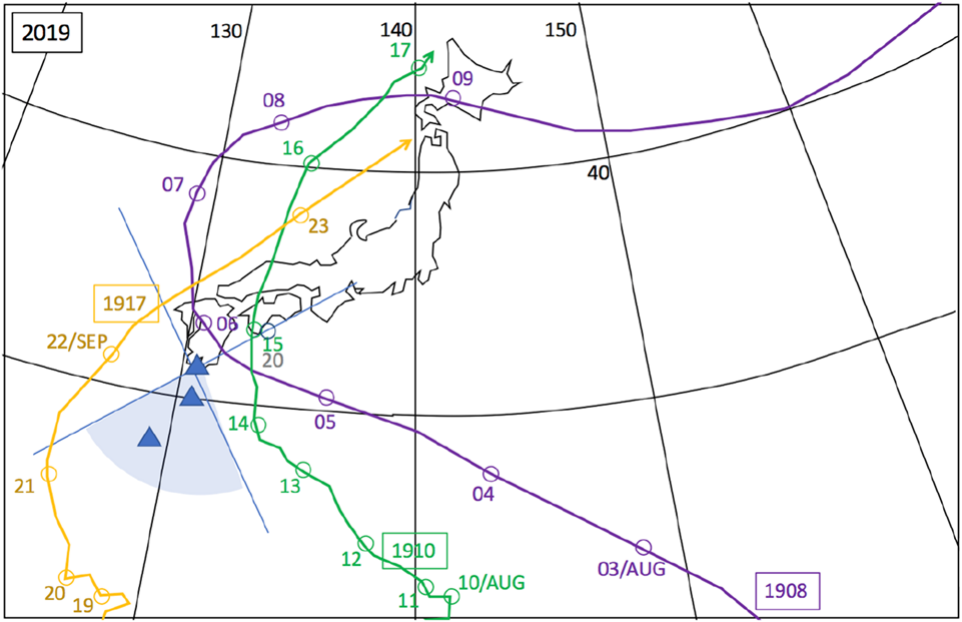
Meteorological history of tropical cyclons T-1908, T-1910, and T-1917^48^. This figure is drawn, same with Fig. 2. HKMT drew the map with Microsoft PowerPoint software and HKMT drew the image with Microsoft PowerPoint software and holds the copyright.

**Figure 4:**
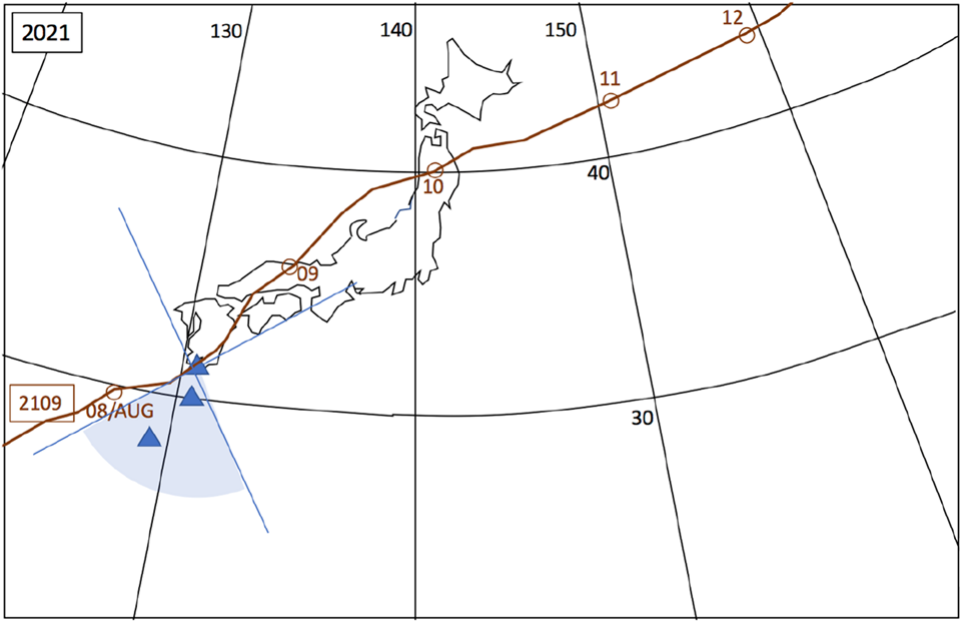
Meteorological history of tropical cyclons T-2109^48^. This figure is drawn, same with Fig. 2. HKMT drew the map with Microsoft PowerPoint software and HKMT drew the image with Microsoft PowerPoint software and holds the copyright.

The observations were conducted in the city of Kagoshima, Japan. In Japan, Kagoshima is known to be a city prone to typhoons. A typhoon is defined as a TC when it develops in the Northwestern Pacific Basin, one of the most active TC areas in the world^47^. All types of typhoons are most common from July through to September in Kagoshima. During the current observations, muographic data were collected during the three previous typhoon seasons in 2016 (July 19-September 19, 2016), 2019 (July 10-October 10, 2016), and 2021 (July 10-October 10, 2016). During these observation periods, eight typhoons (T-1610, T-1612, T-1613, T-1616 in 2016, T-1908, T-1910, T-1917 in 2019, T-2109 in 2021) approached Kagoshima. In 2020, no typhoon landed in Japan. The recorded characteristics of the typhoons are summarized in Table 1. Figures 2, 3 and 4 respectively, show these typhoons' meteorological history in 2016, 2019, and 2021.

**Table 1:** Characteristics of the typhoons during which muographic data were collected^48^.

Year	ID	Minimum pressure (hPa)	Maximum wind speed (m s^−1^)	Maximum strong wind area (km)
2016	T-1610	940	45	SE: 600 NW: 440
2016	T-1612	955	35	E: 170W: 110
2016	T-1613	1000	20	SE: 280 NW: 170
2016	T-1616	930	50	NW: 440 SE: 330
2019	T-1908	970	35	NE: 280 SW: 170
2019	T-1910	965	40	S: 1100N: 560
2019	T-1917	970	35	SE: 700 NW: 440
2021	T-2109	990	20	SE: 600 NW: 330

A strong wind area is defined as an area where the wind speed exceeds 15 m s^−1^^48^.

### Muograph

**Figure 5:**
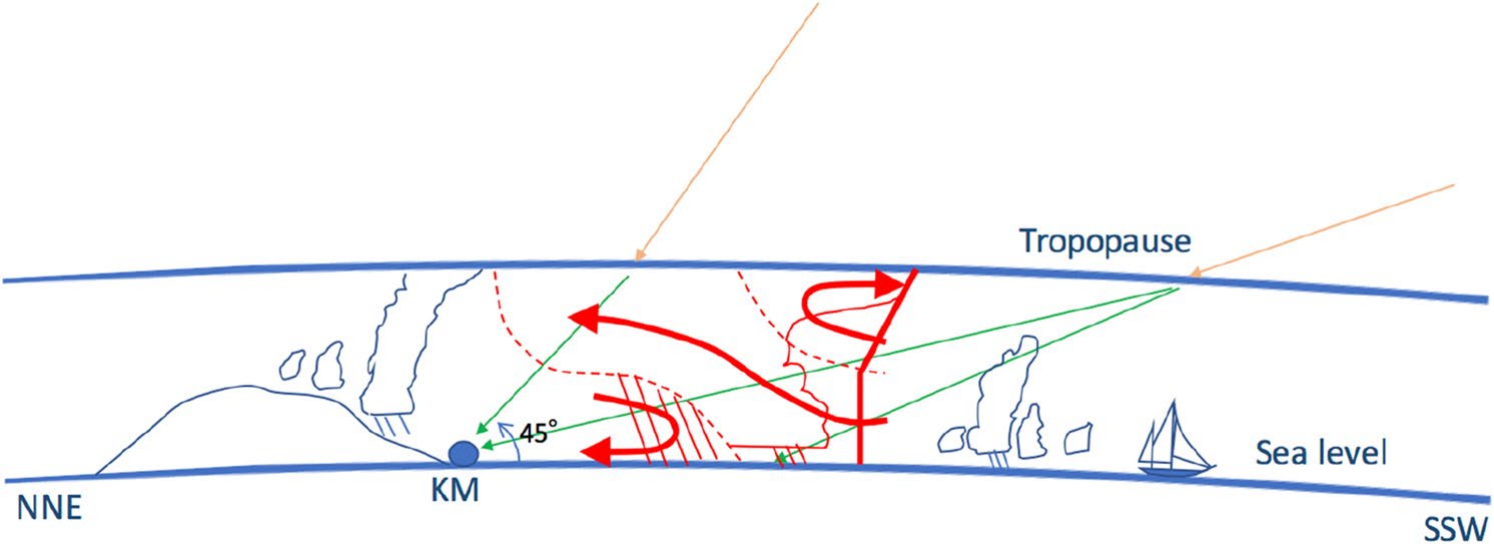
Schematic view of atmospheric muography conducted in this work. Orange and green arrows indicate the galactic cosmic rays (GCRs) and muons, respectively (a single GCR particle can generate numerous muons). The label "KM" indicates the location of the Kagoshima Muograph. A schematic diagram (red dashed lines and arrows) showing the airflow relative to a two-dimensional, steady-state MCS^49^ is also overlaid. HKMT drew the image with Microsoft PowerPoint software and holds the copyright.

A muograph is a device for recording the muographic data. The detection plane of the current muograph located in Kagoshima (Kagoshima Muograph: KM) is positioned vertically in relation to the ground surface. KM consists of three layers of segmented scintillation detectors (SSD) where the distance between the uppermost and lowermost stream detectors was set to be 1.5 m. In the KM 2016 model, each position-sensitive plane consisted of *N*_*x*_ = 14 and *N*_*y*_ = 14 adjacent scintillator strips, forming a segmented plane with 14 × 14 segments. The width of each scintillator strip was 10 cm. Therefore, at the beginning of the observation, each SSD had a 1.4 × 1.4 m^2^ active area with a spatial resolution of 10 cm, but these SSDs were eventually upgraded to 15 × 15 segments; hence it now has a 1.5 × 1.5 m^2^ active area. Each SSD was placed at the same interval. In order to separate muons from other soft components such as electrons, positrons, and gamma rays, a radiation shield with thicknesses of 10 cm lead and 2 cm stainless steel was inserted between each SSD interval. Muon events were selected by choosing events with linear trajectories. While the muons pass through this radiation shield straightforwardly, soft components stop or strongly scatter due to their shorter radiation length (5 mm in Pb and 18 mm in Fe). KM had an elevation viewing angle of 0°–45° and an azimuth viewing angle of ± 45°, and it was pointed in the south by southwest (SSW) direction (sea direction); it had the capability to detect muons also arriving from the opposite direction (where the mountain is located), i.e., north by northeast (NNE), but since the viewing angle in this direction was partially covered by mountains, the NNE data were not considered in this work. Figures 2, 3, 4, 5 indicate the vertical and horizontal viewing angles of KM that is located at an altitude of 146 m above sea level.

In the current muography system, the following procedure has been established and the operation is ongoing in a real-time manner. Every 10 min, the event-by-event data are automatically converted to the track number distribution as a function of azimuth and zenith angles. These track number distribution data are transferred to the central data server located in Kyoto, Japan. The member users can access this server anytime to interactively generate muographs and the time-sequential plots of muon counts. In this work, 24 10-min track number distribution data were added to attain an appropriate statistic level so that we can discuss about percent-order variations in the muon rate.

Most muons are generated at the height of 15–20 km above sea level. Therefore, the KM was designed to record the muons travelling through an atmosphere 20 km to 500 km thick; the length of the muon’s pathway (MPL) to the surface of the Earth depends on its arrival angle (Fig. 5). Due to (a) the detector's geometric acceptance and (b) the zenith angular dependence of cosmic ray muons, 50% of the total muon events were recorded within the zenith and azimuth angular range between 60^o^ and 80° and ± 15°, respectively.

**Figure 6:**
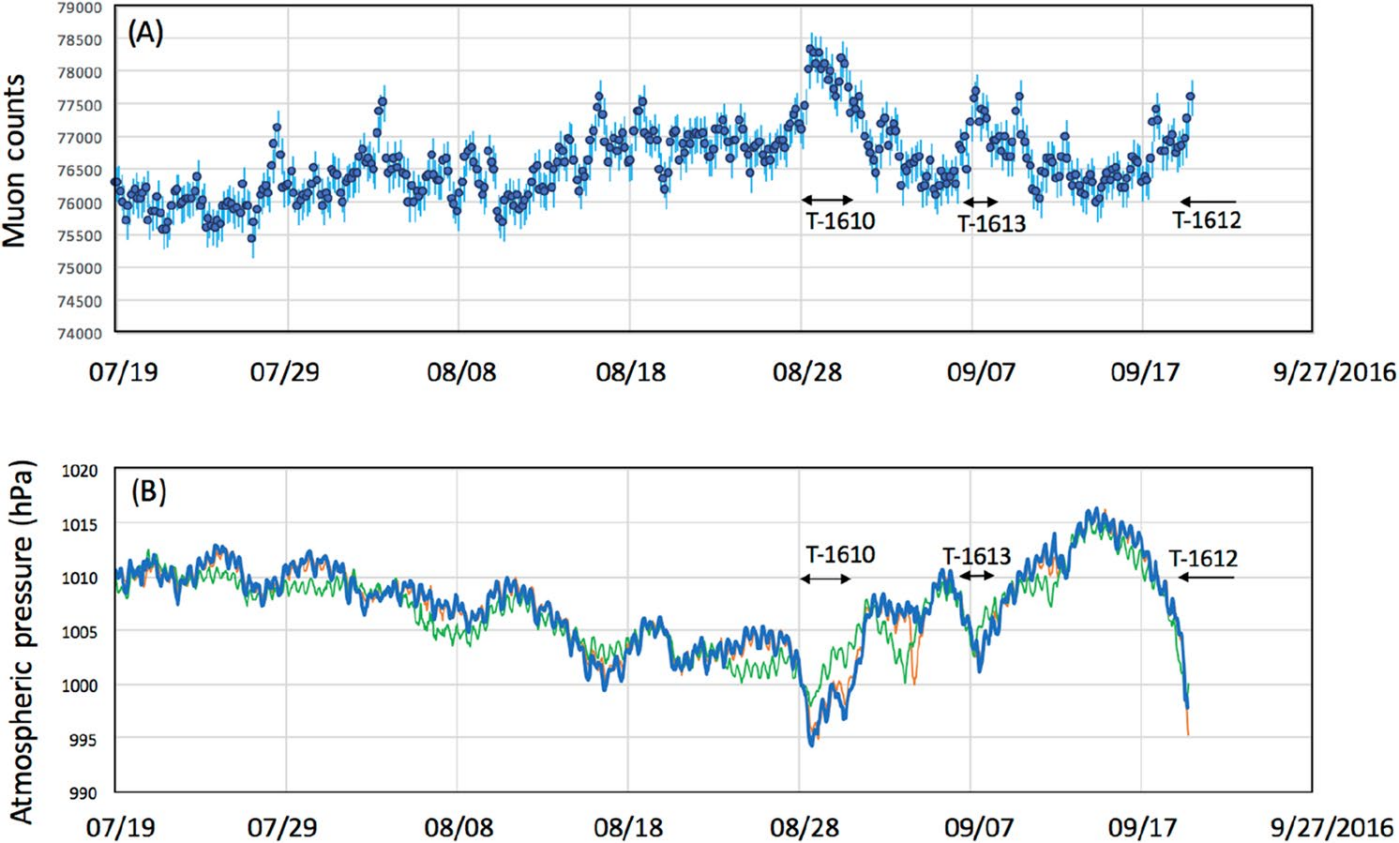
Observed time-dependent variations of the muon count rate in the 2016 typhoon season (**A**). The atmospheric pressure values observed at three BP monitoring stations of Kagoshima (blue), Yakushima (Orange) and Naze (Green)^48^ are shown (**B**). The vertical bars associated with the data points indicate 1σ error bars. Arrows indicate the period that T1610, T1612, and T1613 are added. T1616 is out of the range of the current measurement.

**Figure 7:**
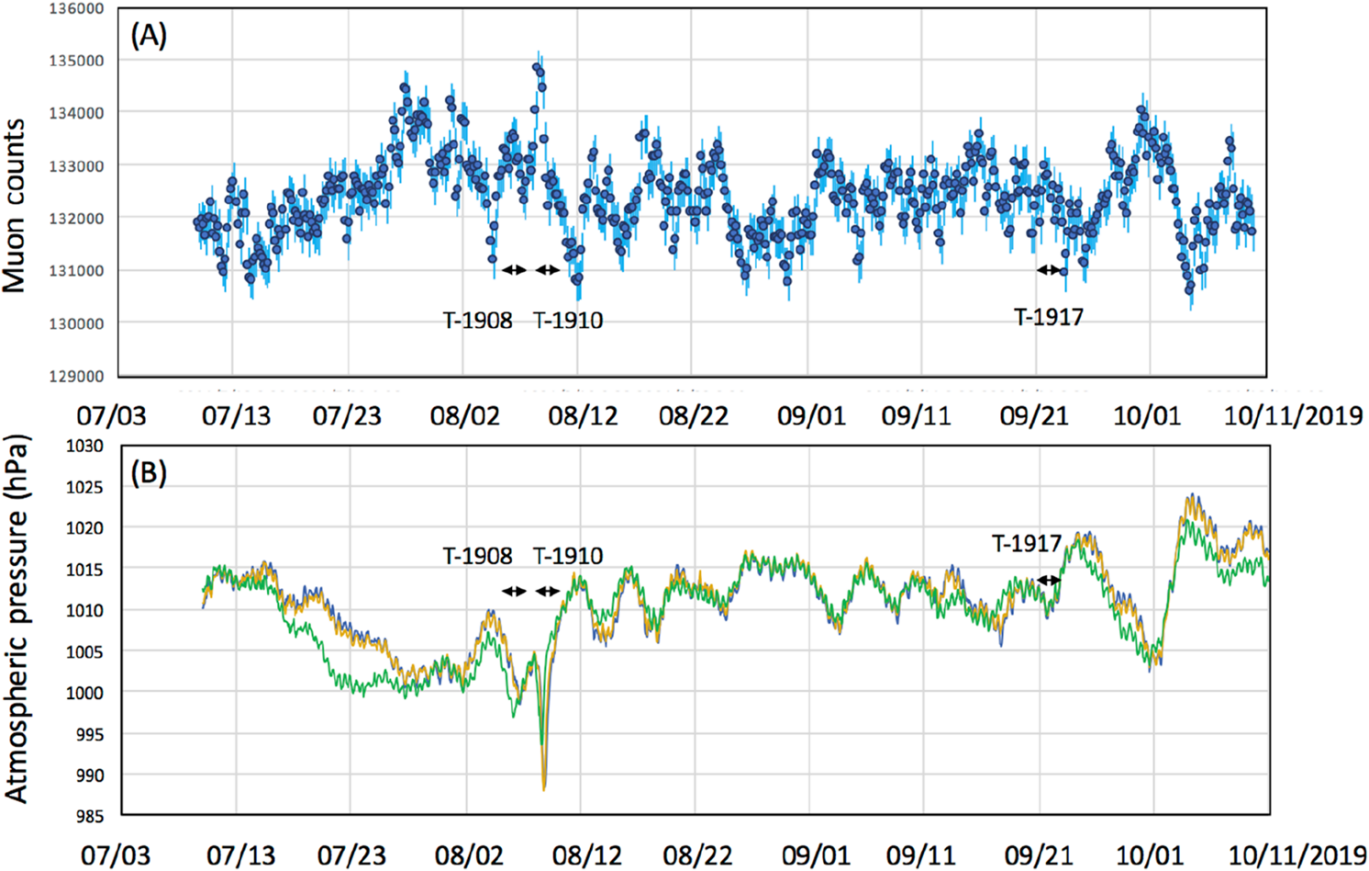
Observed time-dependent variations of the muon count rate in the 2019 typhoon season (**A**). The atmospheric pressure values observed at three BP monitoring stations of Kagoshima (blue), Yakushima (Orange) and Naze (Green)^48^ are shown (**B**). The vertical bars associated with the data points indicate 1σ error bars. Arrows indicate the period that T1908, T1910, and T-1917 approached.

**Figure 8:**
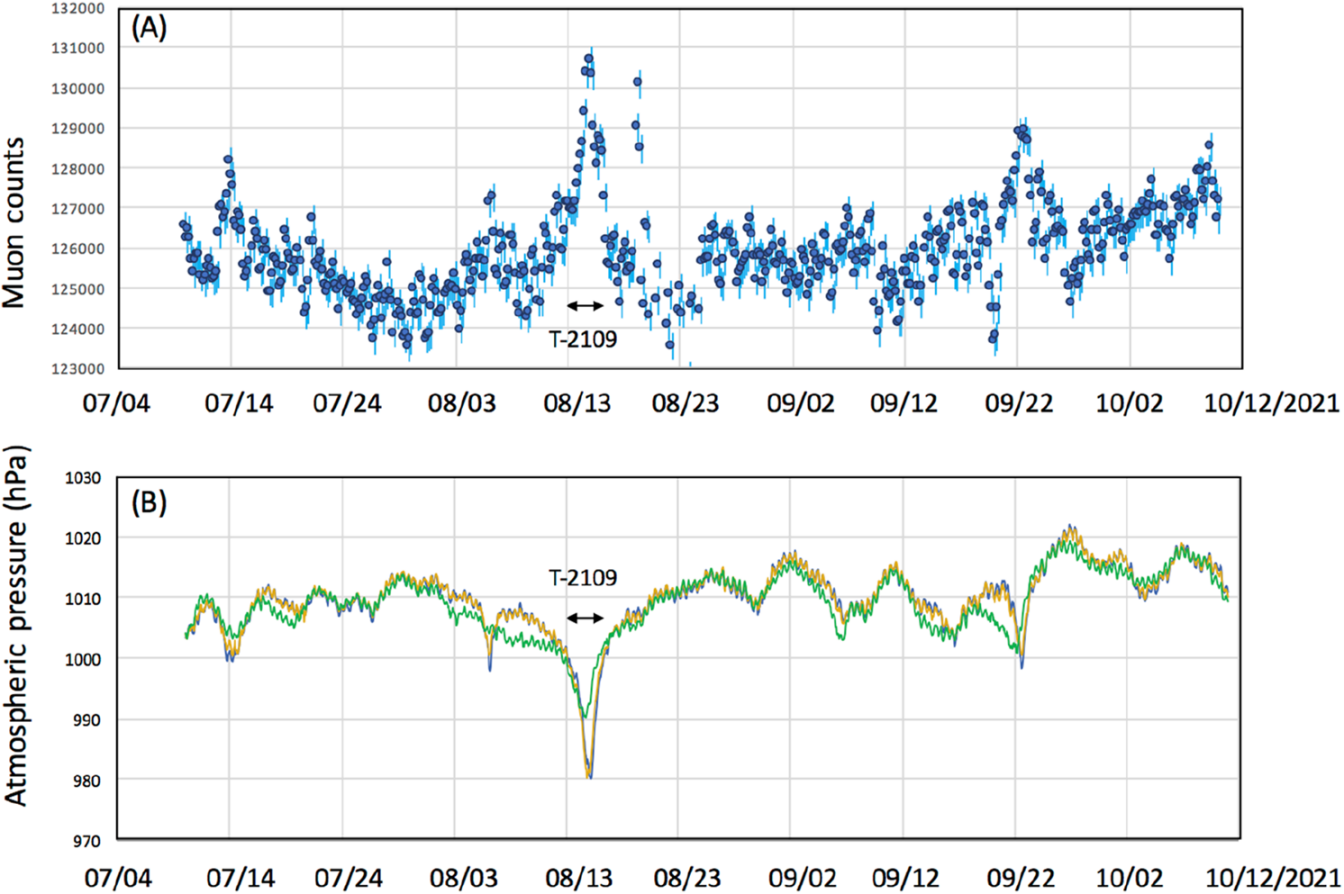
Observed time-dependent variations of the muon count rate in the 2021 typhoon season (**A**). The atmospheric pressure values observed at three BP monitoring stations: Kagoshima (blue), Yakushima (Orange) and Naze (Green)^48^ are shown (**B**). The vertical bars associated with the data points indicate 1σ error bars. Arrows indicate the period that T2109 approached.

Figures 6, 7, 8 compare the muon counts collected every 4 h with the atmospheric pressure values observed at three barometric pressure (BP) monitoring stations (Kagoshima, Yakushima and Naze as indicated in Figs. 2, 3, 4) within the viewing angle of the KM^48^. Yakushima is located 150 km south of Kagoshima, and Naze is located 380 km southwest of Kagoshima. There is a clear anti-correlation between muon counts and BP. Overall, the BP variations are in agreement among these three stations and the muon count rate increases by ~ 2% when the pressure drop (PD) had reached 10 hPa. By considering many of these are slanted muons, this level of the muon flux increase is in agreement with the relationship shown in Fig. 1. As shown in Fig. 6, the PD event, which started on August 28, 2016, was associated with the approach of T-1610; since T-1610 remained south of Kagoshima for a week, the PD steadily continued until the end of August when this typhoon transformed into an extratropical cyclone. The PD associated with the approach of T-1616 started on September 14, and accordingly, an increase of the muon count rate was observed; however, since the path of the typhoon was much closer to Kagoshima, this typhoon caused an electricity blackout, and data taking at the Kagoshima MOS was cut off at 20:00 on September 19th. Two other minor PD events observed within the periods between September 1–4 and between September 6–9 have been respectively associated with T-1612 and T-1613. As shown in Fig. 7, the largest PD event observed within the period between August 10–17, 2019, was associated with T-1910, while the second-largest PD event was observed within the periods between September 20–23 was associated with T-1917. Another PD event observed on August 4–7 was associated with T-1908. In Fig. 8, the largest PD event was observed within the period between August 4–10, 2021 and associated with T-2109. The pressure drops and the increases in muon flux are not all related to TC. In Figs. 6, 7, and 8, several local pressure drops can be seen. However, these drops all didn't reach 1000 hPa. Accordingly, increases in muon flux associated with these drops are also smaller, indicating that these drops are associated with the passage of non-TC low pressures. On the contrary, the pressure drops associated with T1612, T1613, T1908, and T2109 are much larger and they dropped below 1000 hPa, and accordingly larger increases in muon flux can be seen. In the current work, we focused on only these large drops, and the threshold was set to be < 1000 hPa. Life spans of the pressure drops are generally associated with the traveling speed and traveling trajectories of TCs. For example, in the case of TC 1610, the traveling speed during the period between August 21–27 was much slower than other periods. Moreover, this CL stayed in the south of Kyushu Island for a week.

For monitoring the vertical profile of tropic cyclones (TCs), the main source of the observation noise comes from the statistical fluctuation of the muon counts. If more muon counts are recorded, relative statistical fluctuations are reduced in proportional to *N*^0.5^/*N*, where *N* is the muon count. Therefore, in order to improve the observation quality with higher time resolution and with lower observation noise, larger-sized detectors will be needed. TC-1908 also follows the overall trend of the TC-driven PD. In Kagoshima, the pressure drop due to the TC-1908 passage was 10 hPa. Therefore, the expected variations in the muon flux was 2%. The observed reduction in the muon rate was 2% (from 33,500 to 32,800 counts/h). The muon rate also increases, corresponding to the TC-1612 passage. However, more quantitative discussion is difficult, since the muon data taking stopped due to a lightning struck. As can be seen in Figs. 7 and 8, such a data taking termination didn't occur after 2017 since we strengthened the electric system by adding redundant backup power supplies, surge protectors, etc. in the Kagoshima Muography Observatory.

If we neglect modulations of the primaries (e.g., by Forbush decrease) and the stratospheric seasonal temperature variations, variations in muon intensity are mostly dependent on ionization through the air since bremsstrahlung is suppressed by a factor of *m*_*e*_^2^/*m*_μ_^2^. Since the muon flux varies as a roughly linear function (2000–5000 g cm^−2^) of the matter thickness for small variations in average density along the muon paths^43^, the flux-pressure relationship shown in Fig. 1 (1% variations in the muon flux per 5-hPa variations in the atmospheric pressure) is applicable to near horizontal muons (up to ~ 80° from zenith). Variations in the muon flux seen in Figs. 6, 7, and 8 were consistent with the corresponding observed pressure drops. For example, for T-1610, the muon flux increased by 2% that is corresponding to the pressure drop by 10 hPa while the pressure drop observed at Kagoshima meteorological station was also 10 hPa (from 1005 to 995). For another example, for T-2109, the muon flux increased by 4.8% that is corresponding to the pressure drop by 24 hPa while the pressure drop observed at Kagoshima meteorological station was 25 hPa (from 1005 to 980).

## Discussion

As shown in Figs. 6, 7, and 8, the overall trend of the TC-driven PD events recorded at these weather stations matched except for those observed in the period between September 1–4, 2016 (T-1612 PD), and between August 4–7, 2019 (T-1908 PD). The common feature of these discrepancies can be summarized as follows: The PD negative spikes associated with T-1612 and T-1908 can be seen in the Kagoshima and Yakushima pressure data, but these cannot be seen in Naze data, and vice versa. Since the distance between Kagoshima and Yakushima is 130 km, and the distance between Kagoshima and Naze is 360 km, we expected meso-beta scale pressure anomalies during the aforementioned periods. We also anticipated that these anomalies could be captured with KM.

**Figure 9:**
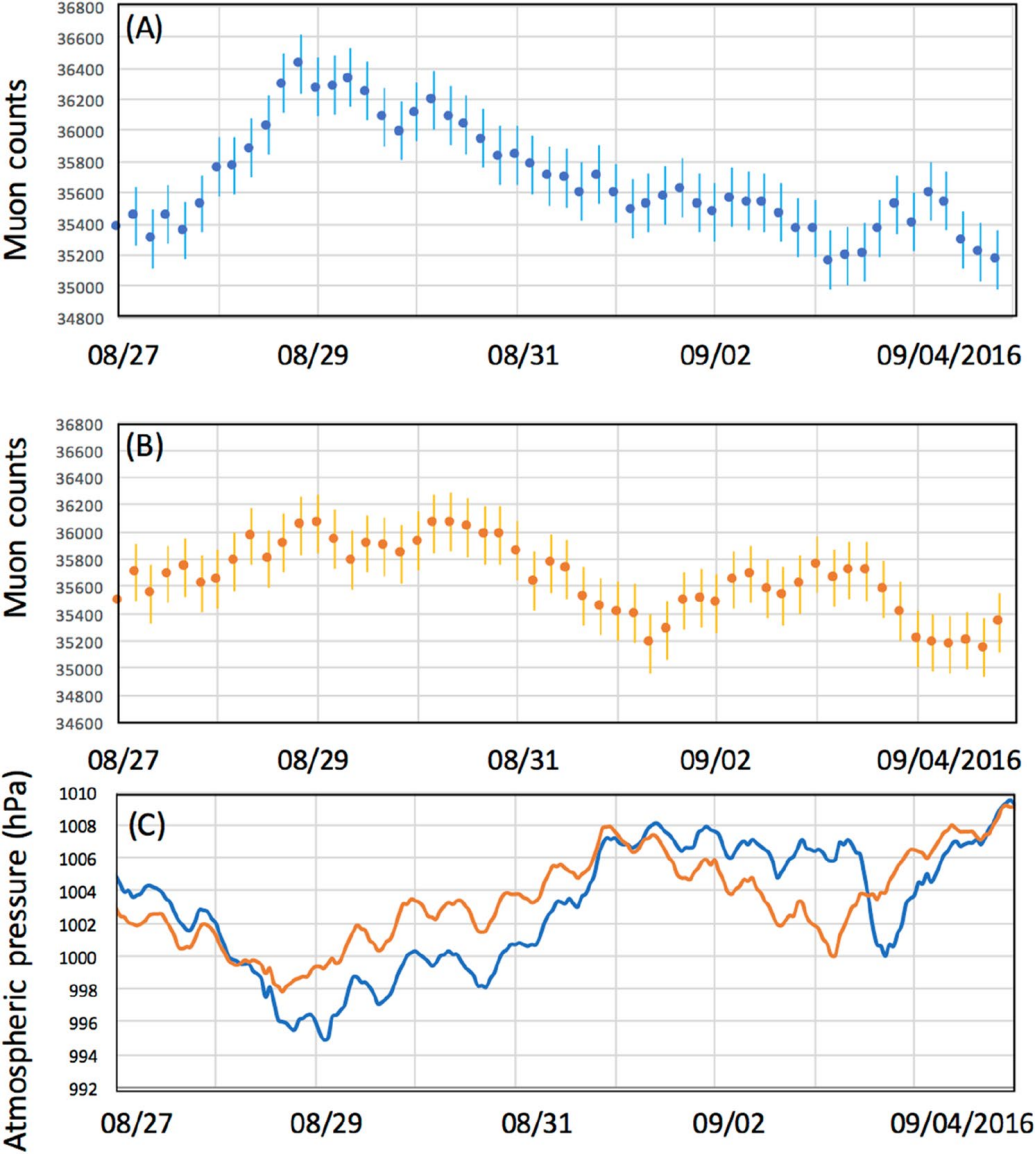
Observed time-sequential muon count rate at high (0.4 < tan *θ* < 0.8) (**A**) and low (0 < tan *θ* < 0.4) (**B**) elevation angles. The vertical bars associated with the data points indicate 1σ error bars. The time-dependent BP variations observed at Yakushima (blue) and Naze (orange) are also shown (**C**). The first pressure drop was associated with the T-1610 passage, and the second pressure drop was associated with the T-1612 passage.

Figure 9 compares the high elevation angle (HEA) (0.4 < tan *θ* < 0.8; 25 km < MPL < 50 km at an altitude of 20 km) (Fig. 9A) and low elevation angle (LEA) (0 < tan *θ* < 0.4; 50 km < MPL < 506 km at an altitude of 20 km) (Fig. 9B) components of the time-sequential muographic data for the 9 days during which T-1610 and T-1612 caused the local PD. The HEA component represents the muons that passed through a more local atmosphere, closer to KM, and the LEA component represents those muons that passed through a less local atmosphere, further from KM. In Fig. 9C, the time-dependent barometric variations observed at Yakushima and Naze stations^48^ are presented for reference. As can be seen in Fig. 9A,B, there are two muon count rate (MCR) increase events between August 27–September 1 and September 1–5. These MCR increase events were interpreted as PD events associated with T-1610 and T-1612. These events were labeled MCR1610 and MCR1612 here. In Fig. 9A,B, we find that the magnitude of HEA-MCR1610 is larger than LEA-MCR1610. Also, we find that the timing and duration are both different between HEA-MCR1612 and LEA-MCR1612. These results are consistent with the pressure variations observed at Yakushima (closer to KM) and Naze (further from KM) stations: PD smaller than that at Yakushima was observed at Naze for T-1610, and at Naze, T-1612 associated PD appeared earlier and lasted longer than Yakushima. Differences between the magnitude of HEA-MCR and the magnitude of LEA-MCR are not always the same. These differences depend on the trajectory, the speed, the size, and the shape of TCs.

In Fig. 9C, we can see that the pressure drop started a couple of day earlier in Naze than the time it started in Yakushima. This is because TC approaced to Naze before it approached to Yakushima. Accordingly, increase in the LEA muon count rate started earlier than increase in HEA muon count rate (Fig. 9B) since the current muography detector was directed towards Naze. This result indicates that if the mugraphy detector is focused towards the same direction of the TC passages, we can detect the far distant pressure drop before the TC approaches to the detector even if there is no meteorological stations in this direction. For the future cyclone early warning system, a multi-directional muography detector that covers all the azimuth angles would be useful.

**Figure 10:**
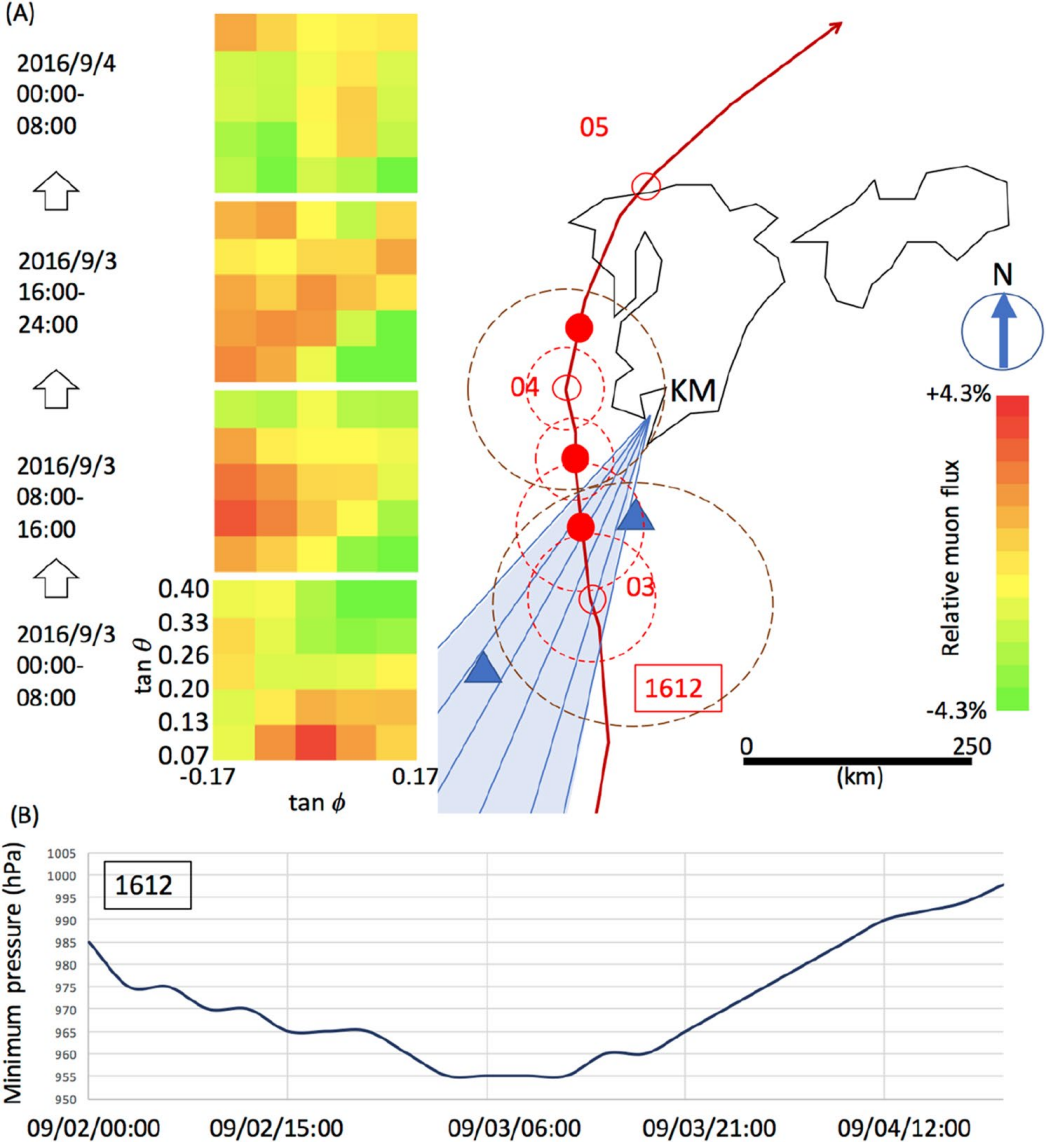
Time-sequential muographic images (**A**). The four images show the muon flux variations observed every 8 h within the period between 00:00 on September 3, 2016, and 08:00 on September 4, 2016. The horizontal width of these muographic images corresponds to the shaded area indicated on the map on the right side. The meteorological history of T-1612 is also shown. Triangular symbols indicate the atmospheric pressure gauge stations (Yakushima Station is in the north, and Naze Station is in the south). The symbol KM indicates the location of Kagoshima Muograph. The red numbers and red hollow circles respectively indicate the date and the position of the T-1612's center. The filled red circles indicate the T-1612 positions at 08:00, 16:00 on September 3, and 08:00 on September 4. The red dashed circles indicate the storm area (wind speed > 25 m s^−1^) at 00:00, 08:00, 16:00, and 24:00 on September 3, and the brown dashed circles indicate the strong wind area (wind speed > 15 m s^−1^) at 00:00 and 24:00 on September 3. The time-dependent variations of the minimum pressure of T-1612 are also shown (**B**). HKMT drew the map with Microsoft PowerPoint software and the image with Microsoft PowerPoint software and holds the copyright.

Figure 10 shows the time-sequential muographic images taken in the period between 00:00 on September 3, 2016, and 08:00 on September 4, 2016, to analyze the pressure variations caused by the passage of T-1612. In order to cancel the KM's geometrical acceptance and the muon's zenith angular dependence, the number of muon tracks in each pixel was divided by that averaged over a period of 6 months (including the period during the passage of T-1612). Moreover, each pixel was mapped within an elevation angular range between tangents 0.067 and 0.4, and an azimuthal angular range between tangents ± 0.17. This elevation angular range is respectively equivalent to the horizontal range between 300 and 50 km at an altitude of 20 km. The overall feature is that the large-flux region indicated in the reddish pixels in Fig. 10 was shifted westwards within this period. This large flux-region was interpreted as the low barometric pressure (BP) region associated with the T-1612 passage, and these time-dependent BP variations are consistent with the trajectory of T-1612.

**Figure 11:**
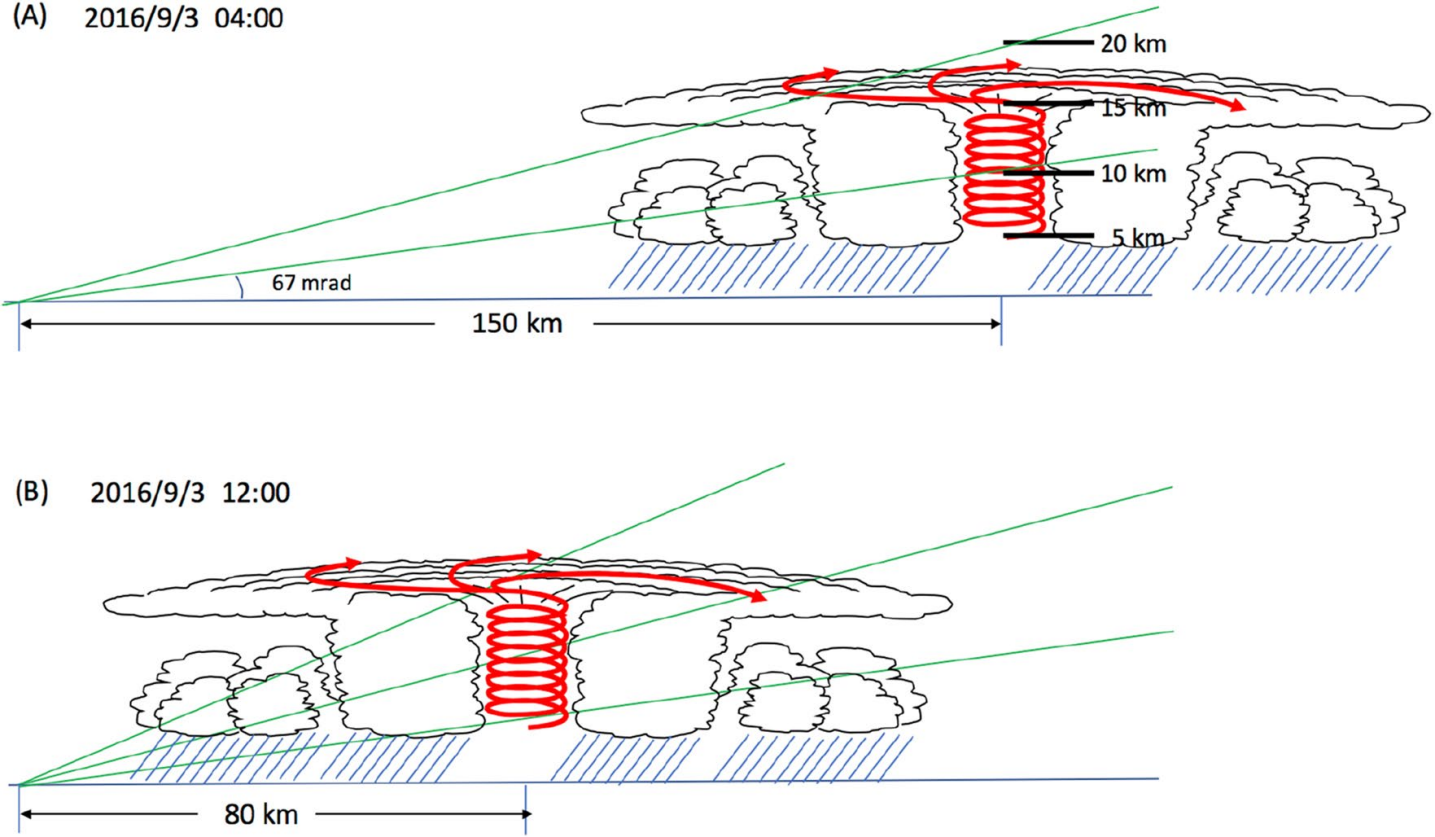
Schematic interpretation of the time-sequential muographic images shown in Fig. 10. The diameter of the storm corresponds to the storm area at 00:00 on September 3. HKMT drew the image with Microsoft PowerPoint software and holds the copyright.

**Figure 12:**
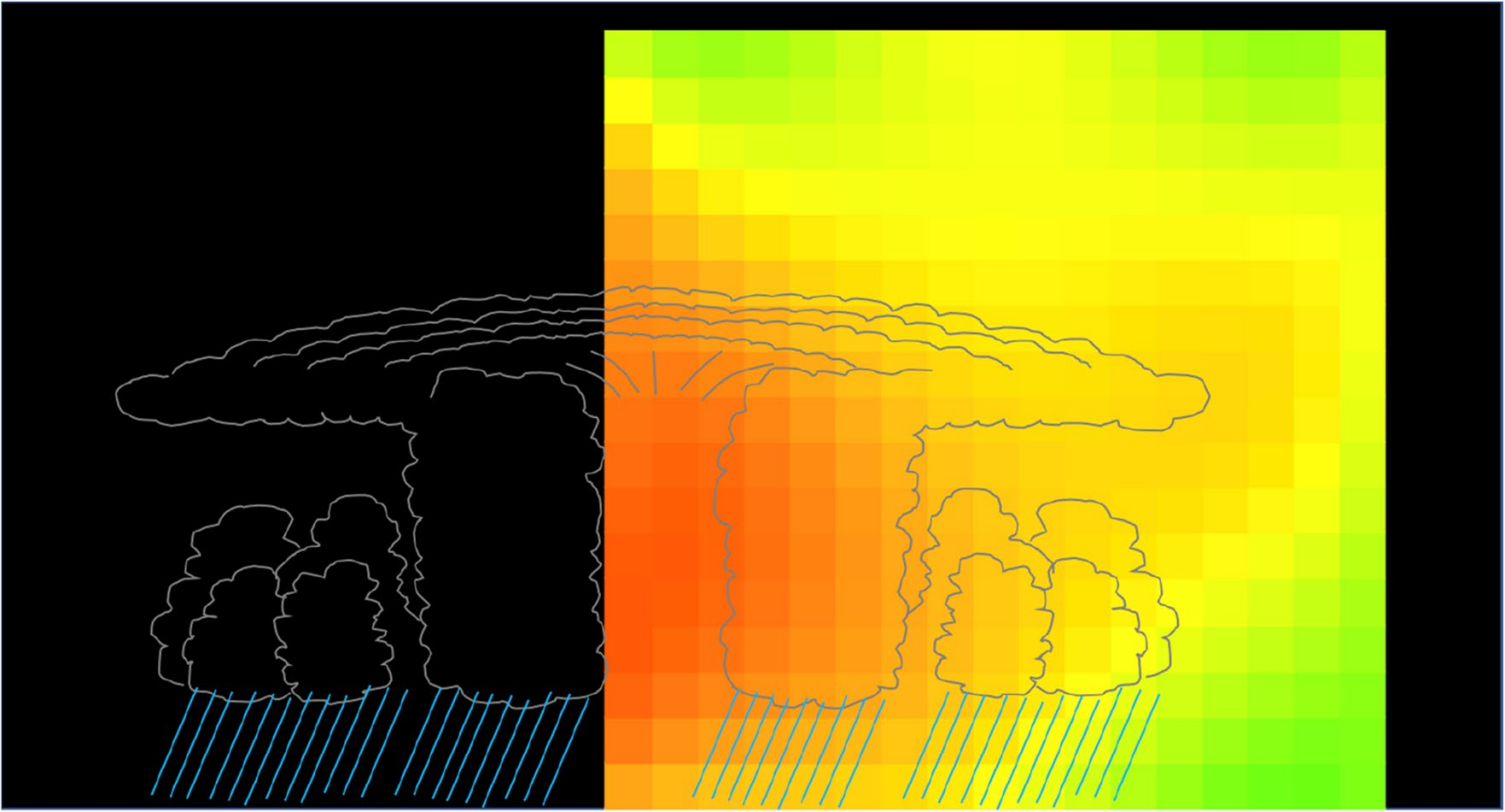
Muographic image of T-1612. The color and angular ranges are the same as in Fig. 10. The position of the centre of the cyclone illustration and its height correspond to the average position and 15 km, respectively. HKMT drew the image with Microsoft PowerPoint software and holds the copyright.

Figure 11 shows the schematic interpretation of the time-sequential muographic images shown in Fig. 10. The distance between KM and the center of T-1612 changed from 180 to 120 km within the period between 00:00 and 08:00 on September 3, 2016. Therefore, the T-1612's lowest-density region where the warm core was located was captured at an elevation angle region between 70 and 130 mrad. T-1612 continued to move northwards after 08:00 on September 3, and as a result, the distance between KM and the center of T-1612 was further shortened from 120 km (at 08:00) to 60 km (at 16:00). As a result, the T-1612’s warm core was captured at an elevation angle region between 130 and 200 mrad. Figure 12 shows the resultant muographic image taken between 08:00 and 16:00 on September 3, 2016. In this figure, the image pixels in Fig. 10 were interpolated with polynomial functions. A low-pressure area that is warmer at its center than at its periphery (warm core) is visualized.

**Figure 13:**
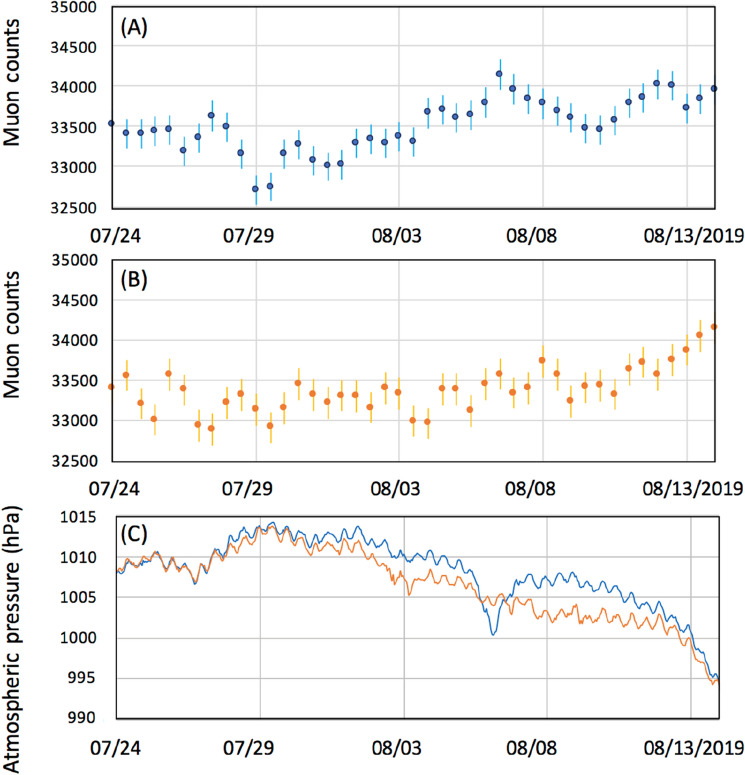
Time-sequential muon count rate observed in the eastern azimuthal angle region (0 < tan *ϕ* < 1.0) (**A**) and the western azimuthal angle region (− 1.0 < tan *ϕ* < 0) (**B**). The vertical bars associated with the data points indicate 1σ error bars. The time-dependent BP variations observed at Yakushima (blue) and Naze (orange) are also shown (**C**).

Figure 13 compares the eastern azimuthal angle (EAA) (0 < tan *ϕ* < 1.0) (Fig. 13A) and western azimuthal angle (WAA) (− 1.0 < tan *ϕ* < 0) components of the time-sequential muographic data for the 21 days during which T-1908 affected the local atmospheric pressure. The EAA component represents the muons that passed through the atmosphere within the directional range between SSE-SSW, while the WAA component represents those muons that passed through the atmosphere within the direction range between SSW-SWW. In Fig. 13C, the time-dependent BP variations observed at Yakushima and Naze stations are presented for reference^48^. As illustrated in Fig. 13A, there is an MCR increase event during the period between August 3–8, 2019. This MCR increase event was interpreted as PD associated with T-1908 and is labeled MCR1908 in the current discussion. However, there is no MCR increase event in Fig. 13B between August 3–8. These results are consistent with that PD which was observed at Yakushima (south of KM within the directional range between SSE-SSW), but it was not observed at Naze (southwest of KM within the direction range between SSW-SWW) stations.

**Figure 14:**
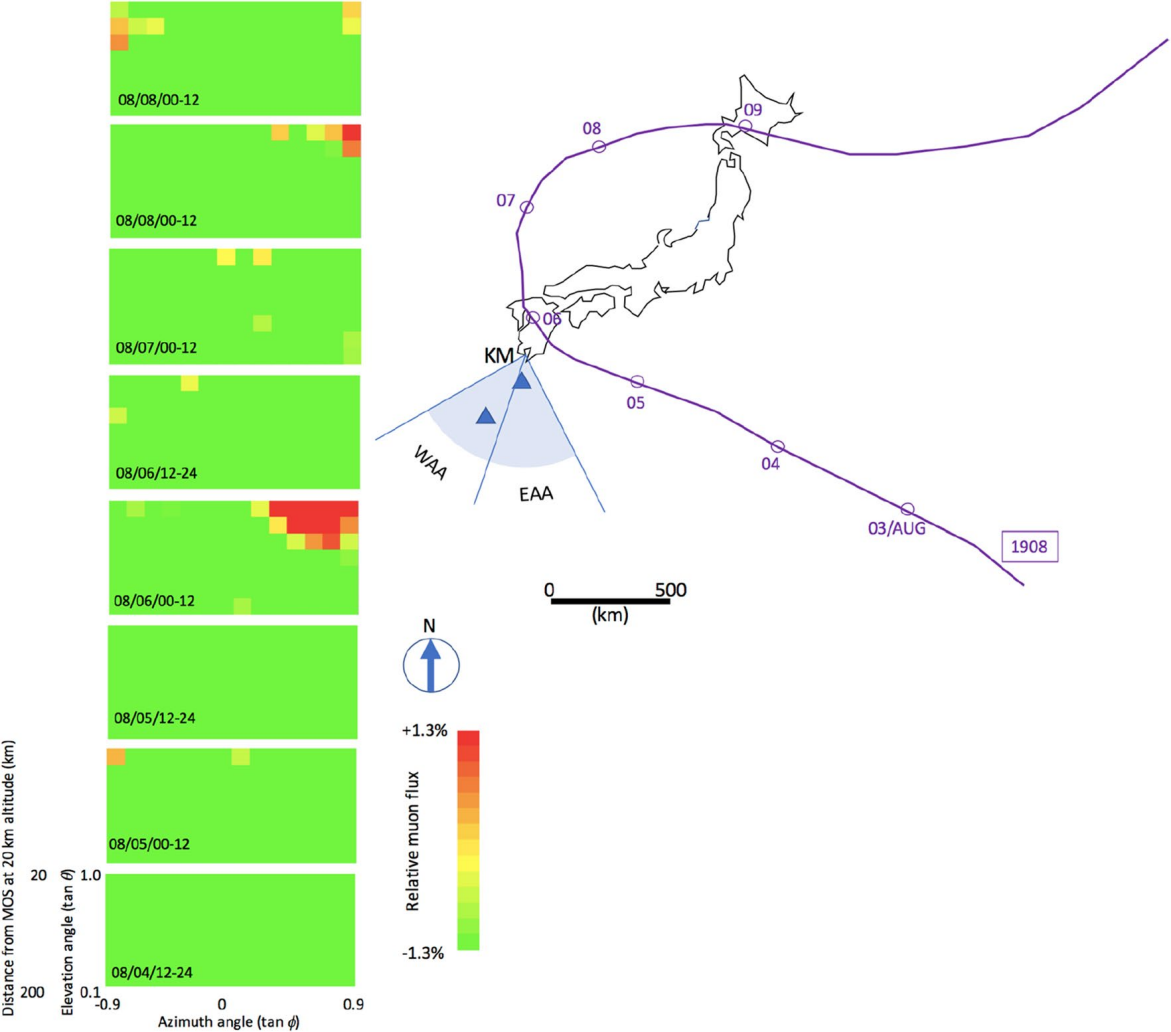
Time-sequential muographic images. The eight images show the muon flux variations observed every 12 h between 12:00 August 4, 2019, and 12:00 August 8, 2019. The horizontal width of these images corresponds to the shaded area indicated on the map on the right side. The meteorological history of T-1908 is also shown. Triangular symbols indicate the BP stations (Yakushima Station is in the north and Naze Station in the south). The symbol KM indicates the location of Kagoshima Muograph. WAA and EAA respectively indicate the eastern azimuthal angle region and the western azimuthal angle region used in Fig. 11. HKMT drew the map with Microsoft PowerPoint software and HKMT drew the image with Microsoft PowerPoint software and holds the copyright.

Figure 14 shows the time-sequential muographic images taken between August 4, and August 8, 2016 to analyze the pressure variations caused by the passage of T-1908. Like previous time-sequential muographic images, the KM's geometrical acceptance and the muon's zenith angular dependence were corrected in this figure. These images were mapped within an elevation angular range between tangents 0–and 1.0, and an azimuthal angular range between tangents ± 1.0. This elevation angular range was respectively equivalent to the horizontal range between 506 and 20 km at an altitude of 20 km. The overall feature was that the large muon flux region indicated in the reddish pixels in Fig. 13 appeared in the HEA-EAA region between 00:00 and 12:00 on August 6, 2019. This large muon flux region was interpreted as the low barometric pressure (BP) region associated with the T-1908 passage, and these time-dependent BP variations are consistent with the trajectory of T-1908.

In conclusion, it was found that an increase in the muon flux is associated with a decrease in pressure associated with the development of TC, indicating atmospheric muography can track density variations associated with TC. Moreover, since muography measures the near-horizontally integrated density of the atmosphere, and BP stations measure the vertically integrated density of the atmosphere, a three-dimensional image of the atmospheric density distribution can be reconstructed with joint inversion of these techniques. Alternatively, the placement of multiple muographs (multidirectional muography) at dispersed locations could also be used to generate a three-dimensional image of the volume within the viewing angle of these muographs. Each muograph unit is low-cost and versatile, so it is possible to position these units nearly anywhere on land. We anticipate that real-time three-dimensional muographic monitoring of TC can become practical and widespread in the near future.

## Method

Kagoshima Muograph (KM) originally consisted of 84 counter bars (3 layers of (*N*_*x*_ + *N*_*y*_ = 14 + 14)), 1 muon readout module, 1 high-voltage power supply (Matsusada Precision HAR-2N300) and 2 radiation shields. Each counter bar consisted of a plastic scintillator strip (Bicron BC-408) connected to a photomultiplier tube (PMT; Hamamatsu H7724) via an acryl lightguide. Six counter bars were eventually added to KM to make 3 layers of (*N*_*x*_ + *N*_*y*_ = 15 + 15). Each radiation shield consisted of a 10-cm thick lead plate covered by a 1-cm thick stainless-steel plate. The PMT signals were discriminated and sent to the field-programmable gate array (FPGA) for logical processing of these signals^50^. The muon track data were sent to a computer every 10 min and subsequently uploaded to the external server so that a remote computer could access the data in real-time.

## Supplementary Information

Supplementary Information.
